# Molecular allergology approach to allergic diseases in the paediatric age

**DOI:** 10.1186/1824-7288-35-29

**Published:** 2009-10-05

**Authors:** Claudia Alessandri, Danila Zennaro, Alessandra Zaffiro, Adriano Mari

**Affiliations:** 1 Center for Clinical and Experimental Allergology, IDI-IRCCS, Rome, Italy

## Abstract

Identification, characterization, and purification of allergens are essential for the structural and immunologic studies needed to understand how these molecules induce specific IgE antibody production by the human immune system. Advances in molecular biology techniques have led to the production of recombinant allergens having constant properties, allowing detection of specific IgE directed against different molecular components of an allergenic source. Presence of homologous allergens in different sources is the reason for cross-reaction. Molecule-based diagnostic tools can lead to better interpretation of poly-sensitizations, observed by ST and in vitro tests using allergenic extracts as they were made before. Some examples IgE sensitization to major genuine allergens and panallergens will be presented.

## 

The Pharaoh's death tale occurred in ancient Egypt vanishes into thin air. It could have been due to anaphylactic shock caused by a bee's sting. Before and after that time and throughout millennia similar events, which happened to other human beings, have not been explained. The era of the diagnosis in allergy began during the last part of the nineteenth century when the skin test (ST) with very raw extracts from presumed allergenic sources were first applied to humans [1]. In 1967 after the discovery of specific IgE in the sera of myeloma patients [2], in vitro tests for allergy diagnosis started to appear on the scene [3]. To date these tests as well the ST are based on allergenic extracts, the same used for specific immunotherapy. The real "Copernican revolution" in the allergy field started during the late eighties, when the first recombinant allergen was cloned; since then allergology and molecular biology joined together, beginning a closer and essential relationship [4].

As far as there is still some confusion on the proper use of some terms, an appropriate terminology is needed in order to follow this review: "allergenic source", "allergenic extract", "allergen" are not interchangeable terms [4].

### Allergenic Source

This term is used to identify the allergenic source material often matching a whole organism (i.e. mite and moulds) sometimes describing some of its tissues (i.e. the dog epithelium, the hen's egg, the cow's milk). In both cases they are allergenic sources, not allergens as they are often defined.

### Allergenic extract

The allergenic extracts normally used in vivo diagnosis (i.e. ST) and in vitro diagnosis (e.g. RAST, ImmunoCAP, Immulite) come from defined allergenic sources as exemplified above. They are obtained by several different buffer extraction procedures, in general optimized to extract most of the unknown proteins from the raw material.

### Allergens and allergenic epitopes

Allergens are proteins or glycoproteins having a molecular weight ranging from 5 to 150 kDa, and an isoelectric point between 4 and 9. Allergenic determinants or epitopes represent the structures recognised by IgE. An allergenic molecule can have linear epitopes making up a specific aminoacid sequence along its primary structure, and conformational epitopes generated by the protein folding. In order to better understand, we can think of an allergenic molecule as a necklace as shown in figure 1: its pearls represent the aminoacids. A linear epitope is made by an aminoacid sequence (i.e. several pearls in a row), and the specific IgE bind to the epitope after its recognition. A conformational epitope is made by aminoacids that are in distant positions in the row but very close each other when the necklace is folded.

**Figure 1:**
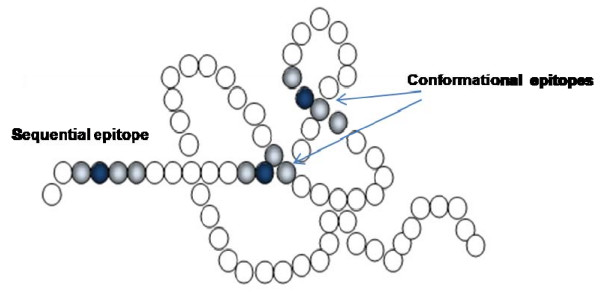
**Conformational and sequential epitopes**.

Molecule foldings are essential for immunological sensitization induction and for the antibodies production. However many allergenic proteins, when exposed to heat or digestion, can lose the conformational epitopes; thus the molecule can be opened and the linear epitopes exposed [5-8]. On these bases a theoretical classification has been established for instance for food allergens: class 1 food allergens and class 2 food allergens [9]. Class 1 food allergens are digestion and heat resistant proteins; they are able to act as sensitizers at the gastrointestinal level. They can give moderate (oral allergy syndrome, OAS) to systemic reactions [10]. Proteins of milk, eggs, fish, peanuts, and some vegetables (containing LTP) are Class 1 food allergens. Most of them are typical childhood allergens (Table 1). Class 2 food allergens are mainly found in vegetables, but can also be present in animal derived foods. They are not resistant to heat and digestion and are generally considered unable to cause systemic symptoms. Sometimes they do not trigger symptoms at all. Patients usually show mild symptoms restricted to the oropharyngeal cavity (OAS), and seem to be primary sensitized by inhalant allergens. The subsequent reaction to plant foods is based on the presence of homologous allergens in both plant allergenic tissues: Bet v 1 proteins, profilins. They are called "non sensitizing elicitors" (Table 2). This phenomenon explains why some patients suffer from reactions, sometimes severe, when they ingest allergenic foods never ate before. Either the aminoacids sequence homology or the presence of a similar epitope patch on the protein surface causes this phenomenon, usually called "cross-reactivity", but better identified as "IgE co-recognition" [11].

**Table 1:** Examples of Class 1 food allergens

**Allergenic Sources**	**Proteins**	**Nomenclature**
**Cow's milk**	Caseins	Bos d 8
	
	Lactalbumin	Bos d 4
	
	Lactoglobulin	Bos d 5

**Egg's white**	Ovomucoid	Gal d 1
	
	Ovoalbumin	Gal d 2

**Peanut**	Vicilin	Ara h 1
	
	Conglutin	Ara h 2
	
	11S Globulin	Ara h 3

**Fish**	Parvalbumin	Gad c 1
		
Cod		

**Plants**	Lipid Transfer Protein	
		
Apple		Mal d 3
		
Peach		Pru p 3

(more details on )

**Table 2:** Examples of Class 2 food allergens

**Allergenic Sources**	**Proteins**	**Nomenclature**
Apple	Bet v 1-like proteins	Mal d 1
		
Peach		Pru p 1
		
Celery		Api g 1
		
Hazelnut		Cor a 1

Banana	Chitinases	Mus a Glucanase
		
Kiwi, golden		Act c Chitinase
		
Kiwi, green		Act d Chitinase
		
Tomato		Lyc e Chitinase

Tomato	Profilins	Lyc e 1
		
Apple		Mal d 4
		
Peach		Pru p 4
		
Celery		Api g 4
		
Hazelnut		Cor a 2

(more details on )

It is not always possible to establish *a priori *which, among two or more allergens, has been able to induce the IgE sensitization (sensitizer) or the cross reactivity (elicitor).

Factors needed for proteins cross reactivity are:

1) the cross reactive portion of the epitopes;

2) the cross reactive portion of the IgE (paratope);

3) the affinity among the IgE and the two epitopes on the two related allergens.

Antibody antigen recognition requires 6-8 aminoacids placed on the linear sequence; two different proteins must be at least 35% identical to determine cross reactivity [12], but this should not be a rigid rule as there are several exceptions.

### Diagnosis with molecular allergens

In recent years molecular allergens have been produced and commercialized. They have been obtained as highly purified molecules by either recombinant DNA methods, or by biochemical purification from natural sources. The availability of highly pure allergenic molecules is easing the production which can be standardized and large amounts of allergens can be produced.

In the case of the recombinant allergens, it is possible to introduce specific site mutations to generate hypoallergens, and to clone isoforms.

It is not the purpose of this review to explain in details how to perform specific IgE determinations towards allergenic extracts or allergens. However, it is enough to remind that singleplex diagnostic tests (one result for a single serum specimen [13]) are the same as those used for the IgE determinations towards allergenic extracts, the difference being that the antigen is a highly purified molecule, either natural or recombinant. Currently, using multiplex diagnostic tests (several results for a single specimen [13]) is possible to detect the presence of specific IgE to 103 allergenic molecules, using 20 μl of serum. Each assay is in triplicate, meaning that is repeated three times for each single protein (ISAC system, VBC-Genomics, Austria) [14]. The total cost of one of these tests is lower than the sum of the single determinations carried out with a system in singleplex aiming to test an equal number of allergens (Mari A et al. manuscript submitted).

Molecular allergens have shown a sensitivity greater than 80% in mimicking the allergenic sources [15]; such sensitivity becomes proportionately higher by putting together all single allergenic proteins coming from a single organism or tissue [15-17]. In order to understand the results provided by these tests, it is important to know the clinical and immunological meaning of the tested allergenic molecules. The nomenclature and the classification of the main allergens is herein reported; for more detailed information the Allergome web site  is suggested [18].

### Allergen nomenclature and classification

Allergen nomenclature has been officially defined by the International Union of Immunological Societies Allergen Nomenclature Subcommittee and reported in several documents [19-21] and currently updated and displayed on the official web site: . The allergen nomenclature uses the first three letters from the organism genus name, followed by a single letter (sometimes two) from the species name, and a number indicating the order of identification of the allergen: e.g. Bet v 1, Bet (genus: *Betula*) v (species: *verrucosa*) 1 (order of identification, sometimes homologous groups). Exceptions to this format and other definitions for isoforms, variants, peptides, genes are given in the most updated document [21]. Definitions for isoforms and variants are reported in Appendix 1 as they play a critical role in current diagnostic and future immunotherapeutic use of allergens.

Allergen subsets can also be defined on the basis of the clinical phenotype they can induce:

(a) "*genuine allergens*": proteins contained in a defined allergenic source and those taxonomically closely related: Ole e 1 is the allergen of the olive pollen, able to cross react with other group 1 *Oleaceae *allergens (i.e. Fra e 1); Phl p 1 and Phl p 5 represent the markers of grass pollen group 1 and 5, and Cup a 1 is the allergen of Cupressaceae group 1 (cedar/cypress/juniper); Par j 1, Par j 2 are allergens of pellitory pollen; Der p 1, Der p 2, Der f 1, Der f 2 are named genuine dust mite allergens [22].

(b) *Panallergens *are proteins with a high similarity but not identical, present in different botanical or zoological families, related and unrelated by taxonomy [23-27].

The panallergen distribution is broader than for genuine allergens, explaining why they are not always uniformly IgE recognized by 100% of the patients sensitized to them.

Several panallergen groups have been described up to now:

1) **Bet v 1-like proteins **(Fagales-related proteins), widely distributed in the plant kingdom, are defence proteins, belonging to the family of "pathogenesis-related proteins" or PR-10; they are present in tissues devoted to reproduction (pollen, seeds, and fruits) [28]. They are molecules not resistant to heat or digestion, and produced according to the degree of maturity of plants and can cause symptoms by both inhalation and ingestion. The Bet v 1-like proteins are present, in addition to the pollen of birch, also in pollen of the *Fagales *family (i.e. beech, oak, chestnut, and hazel). These trees have different pollination season, causing in certain geographical areas the persistence of symptoms by inhalation (i.e. rhino-conjunctivitis, asthma) for several months, from January to June. In central and southern regions of Italy, where the birch does not spontaneously grow because of the climate, homologous species are present (hazel, oak, beech, chestnut). They can cause sensitization to the same allergen group as birch pollen. The correct understanding and interpretation of cross reactivity within the above described species it possible taking into account their molecular relationship [29]. Furthermore, patients allergic to birch pollen suffer from allergic reactions when they eat various types of fruit and vegetables sharing cross reactivity between Bet v 1 and homologous allergens. These homologous allergens are present in *Rosaceae *(i.e. apple, pear, peach and some nuts), *Apiaceae *(celery, carrot), *Fabaceae *(soya beans, peanuts). Symptoms caused by ingestion of Bet v 1-like proteins contained in food are generally mild (class 2 food allergens). The OAS is an example, though more severe reactions have been reported for soya [30-32]. To date 48 Bet v 1-like allergens have been described.

2) **Profilins **are proteins present in all eukaryotic cells. Those belonging to the plant kingdom have sequence homology higher than 75%. 10-20% of pollen sensitized patients (grass, birch, olive, pellitory) have IgE to profilins [33]. When that happens the cross reactivity between pollen, vegetables and latex is extremely high. Profilins may cause the same symptoms via inhalation, contact, ingestion as the Bet v 1-like proteins do (class 2 food allergens) [34] though severe reactions caused by profilin exposure have never been reported. To date 97 profilins have been described as allergens.

3) The members of most ubiquitous family of Calcium Binding Protein, also defined polcalcins to distinguish them from other calcium binding protein, are pollen components from trees (Bet v 4, Aln g 4, Ole e 3, Jun o 4), grasses (Cyn d 7, Phl p 7) and weeds (Bra n 4, Bra n 7, Bra r 4, Bra r 7, Par j 4).

They are cross-reactive allergens present in pollen. No significant cross-reactivity has been found among the fish parvalbumins and pollen calcium binding proteins [28]. Polcalcins are not present in edible parts of plants. Phl p 7 and Bet v 4 are considered markers of this group. The extended allergen dispersal period caused by the polcalcin release from many different pollen species (Table 3) can cause persistent allergic respiratory symptoms not related to flowering of a single allergenic source [27,35,36]. Severe asthma symptoms have been reported for this group of molecule so far [33]. To date 39 polcalcins have been described as allergens.

**Table 3:** Polcalcins

**Allergens**	***Species***	**Common name**
**Aln g 4**	*Alnus glutinosa*	Alder

**Amb a 10**	*Ambrosia artemisiifolia*	Ragweed
		
**Amb a 9**		

**Art v 5**	*Artemisia vulgaris*	Mugwort

**Bet v 3**	*Betula verrucosa*	Birch
		
**Bet v 4**		

**Bra n 4**	*Brassica napus*	Rapeseed
		
**Bra n 2**		

**Bra r 4**	*Brassica rapa*	Turnip
		
**Bra r 7**		

**Che a 3**	*Chenopodium album*	Goosefoot

**Cup a 4**	*Cupressus arizonica*	Cypress

**Cyn d 7**	*Cynodon dactylon*	Bermuda grass

**Jun o 4**	*Juniperus oxycedrus*	Cedar

**Ole e 3**	*Olea europea*	Olive tree
		
**Ole e 8**		

**Ory s 7**	*Oryza sativa*	Rice

**Phl p 7**	*Phleum pratense*	Timothy Grass

**Syr v 3**	*Syringa vulgaris*	Common Lilac

(more details on )

4) **Lipid Tranfer proteins (LTP) **are true panallergens with a variable degree of cross-reactivity. They are defence plant proteins against the attack of bacteria, fungi, and viruses. This has led them to be ubiquitously expressed throughout the plant kingdom [28]. The highest expression levels have been found in peripheral cells associated with cell wall and cuticle of epidermal tissues. Due to their resistance to pepsin digestion and to heat, LTP are considered food allergens which might cause severe reactions (class 1 food allergens). After ingestion, but occasionally also by inhalation or contact, symptoms include all the clinical pictures described for a food IgE mediated reaction up to now, starting from OAS to gastrointestinal symptoms characterized by violent intestinal cramps, or to urticaria-angioedema syndrome, up to anaphylaxis. Our data, collected in a population of more than 30,000 patients, shows that LTP allergy can be present at any age. A relevant number of this patients experience fruit allergy without linked pollinosis (Mari A et al., manuscript in preparation). Cross-reactivity among allergenic LTP present in food has been described, even between members of botanically unrelated species (Table 4). There are no longitudinal studies establishing whether a patient will be monosensitized all lifelong or will develop multiple sensitizations within this group of panallergens. However, sensitization (detection of specific IgE) does not mean clinical allergy (e.g. symptoms on exposure), and fruits or vegetables containing LTP should be avoided only if important symptoms occur after current exposure. The concentration of LTP in fruit varies depending on the molecule localization. Patients sensitized to peach LTP eating peach once peeled are reported. To date 36 LTP acting as allergens by ingestion have been described.

**Table 4:** Vegetables containing LTP

**Allergens**	**Food**	**Allergens**	**Food**
**All c 3**	Onion	**Lyc e 3**	Tomato

**Ara h 9**	Peanut	**Mal d 3**	Apple

**Aspa o 1**	Asparagus	**Ory s 14**	Rice

**Bra o 3**	Broccoli	**Pru ar 3**	Apricot

**Bra r 3**	Turnip	**Pru av 3**	Cherry

**Cas s 8**	Chestnut	**Pru d 3**	Plum

**Cit l 3**	Lemon	**Pru du 8**	Almond

**Cit r 3**	Mandarin	**Pru p 3**	Peach

**Cit s 3**	Orange	**Pun g 3**	Pomegranate

**Cor a 8**	Hazelnut	**Pyr c 3**	Pear

**Dau c 3**	Carot	**Ros r 3**	Rose

**Fra a 3**	Strawberry	**Rub i 3**	Raspberry

**Hel a 3**	Sunflower	**Tri a 14**	Wheat

**Hor v 14**	Barley	**Tri s 14**	Spelt

**Jug r 3**	Walnut	**Vit v 1**	Grape

**Lac s 1**	Lettuce	**Zea m 14**	Corn

(more details on )

5) **Seed storage proteins **make the required nutrients available to plant seeds during sprouting. They include groups of proteins such as the 11S globulins, 2S albumins and the 7S vicilins, widely present in the majority of allergenic seeds: mustard, walnuts, sesame, castor bean, cashew, pistachio, peanuts [12,28]. They are resistant to cooking and digestion (class 1 food allergens). Seed storage proteins are first among the food allergens to be responsible for severe anaphylactic reactions in adults. In the child instead, they are ranked third following milk and egg allergens. To date 66 seed storage proteins acting as allergens have been described.

6) **Tropomyosins **regulate muscle contraction in invertebrates. Exposure to the allergenic sources containing these proteins occurs on a daily basis, as these proteins are widespread in animal species. They have been identified as inhalant allergens (mites, cockroaches) and as food allergens (crustaceans, molluscs, and a fish parasite *Anisakis simplex*) (Table 5). Their presence in the house dust is proportional to the quantity of arthropods infesting house (mites, spiders, silverfishes, cockroaches) [37]. Tropomyosins retain their IgE binding ability even after prolonged heating or gastric proteolysis. The frequent cross-sensitization among different allergenic sources is due to the highly conserved tropomyosin sequences. Running the diagnostic tests with allergenic extracts might lead to positive results for mites, cockroaches, and shellfishes, since these panallergens are contained in all those sources. Missing the IgE molecular profile, it will be impossible to find out whether the mite allergic patient is sensitized to this type of allergen (Der p 10) or to the genuine one from the dust mites (Der p 1, Der p 2). To date 111 tropomyosins have been described as allergens. Eleven tropomyosins are IgE-binding proteins in parasites, mainly nematodes.

8) **Parvalbumins **represent a large group of proteins involved in muscular contraction controlling calcium flow in the muscular sarcoplasm. It has been demonstrated that they are present in white muscle of many fish species; thus, cross-reactivity among different fish species do exist. However, patients allergic to some fish can sometime ingest some other species without risk of allergic symptoms [38,39]. Parvalbumins from fishes and frogs are major food allergens eliciting IgE responses in most fish-allergic individuals [40-42]. Resistance to boiling and enzymes of the gastrointestinal tract may thus allow this allergen to sensitize patients. To date 39 parvalbumins acting as allergens have been described.

**Table 5:** Tropomyosins

**Allergens**	**Sources**		
**Pen i 1**	*Fenneropenaeus indicus*	Shrimps	Crustaceans
		
**Pen a 1**	*Farfantepenaeus aztecus*		
		
**Pan s 1**	*Panulirus stimpsoni*		
		
**Met e 1**	*Metapenaeus ensis*		
	
**Hom a 1**	*Homarus americanus*	Lobster	
	
**Cha f 1**	*Charybdis feriatus*	Crab	

**Ani s 3**	*Anisakis simplex*	Worms	Nematode
		
**Asc s 3**	*Ascaris lumbricoides*		

**Bla g 7**	*Blattella germanica*	Cockroaches	Insects
		
**Per a 7**	*Periplaneta americana*		
		
**Per f 7**	*Periplaneta fuliginosa*		
	
**Chi k 10**	*Chironomus kiiensis*	Midge	
	
**Lep s 1**	*Lepisma saccharina*	Silverfish	
	
**Dro m 7**	*Drosophila melanogaster*	Fly	

**Blo t 10**	*Blomia tropicalis*		Mites
		
**Der f 10**	*Dermatophagoides farinae*		
		
**Der p 10**	*Dermatophagoides pteronyssinus*		
		
**Der g 10**	*Dermanyssus gallinae*		
		
**Lep d 10**	*Lepidoglyphus destructor*		
		
**Tyr p 10**	*Tyrophagus putrescentiae*		

**Cra g 1**	*Crassostrea gigas*	Oyster	Molluscs
	
**Hal d 1**	*Haliotis diversicolor*	Abalone	
	
**Hel as 1**	*Helix aspersa*	Snail	
	
**Tur c 1**	*Batillus cornutus*	Mussels	
		
**Myt e 1**	*Mytilus edulis*		
		
**Myt g 1**	*Mytilus galloprovincialis*		
	
**Oct v 1**	*Octopus vulgaris*	Octopus	
	
**Mim n 1**	*Mimachlamys nobilis*	Clams	
		
**Per v 1**	*Perna viridis*		
	
**Sep l 1**	*Sepioteuthis arctipinnis*	Golden Cuttlefish	

(more details on )

9) Chitinases and glucanases

The outstanding relevance of molecular diagnosis is further exemplified by the understanding of the latex allergic patient profiling. The IUIS web site lists 13 natural rubber latex allergens characterized at the molecular level. Hev b 1, rubber elongation factor, Hev b 3, small rubber particle protein, Hev b 4, microhelix component, Hev b 6, prohevein/hevein, and Hev b 5 (structural protein) are the major allergens recognized by latex-sensitized patients [43,44]. A significant proportion (close to 30-50%) of the patients experiencing allergy to latex shows signs of associated hypersensitivity to various fresh fruits (avocado, banana, chestnut, passion fruit, papaya, tomato, mango, bell-pepper, potato and kiwi) containing homologous molecules without previous sensitization to the fruit proteins. [45-47]. The main molecular allergens responsible of this syndrome are: Hev b 2 (β-1,3-glucanase) [48], Hev b 7 (patatin-like protein) [49], Hev b 8 (Profilins) [44], and overall Hev b 6 (hevein-like domain) [48] (Table 6).

**Table 6:** Allergens linking latex allergy to fruit reactivity

**Sensitizer**			**Elicitors**	
Source	Allergen	Common names	Sources	Name

Latex	Hev b 2	Glucanase	Olive tree	Ole e 9
			
			Bell Pepper	Cap a Glucanase
	
	Hev b 6	Hevein-like Domain	Turnip	Bra r 2
			
			Obeche	Trip s 1
			
			Banana	Mus xp Chitinases
				
				Mus xp Hevein
			
			Acerola	Mal g Hevein
			
			Avocado	Pers a 1
				
				Pers a Hevein
	
	Hev b 11	Chitinases Class 1	Kiwi	Act c Chitinase
			
				Act d Chitinase
			
			Tomato	Lyc e Chitinase
			
			Chestnut	Cas s 5
	
	Hev b 7		Potato	Sola t 1
	
	Hev b 8	Profillin	Mugwort	Amb a 8
			
			Birch	Bet v 2
			
			Bell Pepper	Cap a 2
			
			Goosefoot	Che a 2

(more details on )

Hev b 2 (PR-2 type proteins) are enzymes widely distributed in plants included into pathogenesis-related protein (proteins able to improve the defence mechanisms of plants against pathogens) [50], produced by several vegetable organs such as seeds, roots, leaves, fruits. The major allergens of latex, Hev b 2 (1,3-β glucanase) has been associated with hypersensitivity to foods, especially avocado, banana, chestnut, fig, and kiwi [51,52] and patatin from potato, showed IgE cross-reactivity with bell-pepper [53] and potato [49].

Hev b 6 prohevein (Hev b 6.01) and hevein (Hev b 6.02) has structural homologies with some cereal lectins [54] and other plant lectins and chitin-binding proteins [55].

*Chitinases *(class I chitnases, PR-3 type proteins) are defence enzymes against moulds and insects [56] acting towards chitin that is a structural component of the exoskeleton of insects and the cell walls of most fungi. They contain a hevein N-terminal domain of about 40 amino acid residues with putative chitin binding properties. Their allergenic activity is inactivated by heat whereas enhanced by artificial fruit ripening [57]. Chestnut (Castanea sativa), avocado (Persea americana) [46,58] and banana [59] belong to this group. Another family of chitinases (PR- 4 type proteins induced in potato, tobacco and in turnip (Brassica rapa) in response to wounding, lacks the N-terminal hevein domain [60,61]. The latex allergenic proteins represent the sensitizing allergens, whereas the fruit chitinases are the elicitors (class 2 food allergens).

### Disadvantages of using allergenic extracts

An allergenic extract is commonly used for ST or for in vivo specific IgE determination or for specific immunotherapy. Allergenic extracts have some disadvantages that are important to know:

#### a) Allergen loss

Some fruit proteins are soluble in acid or in basic solution: the extraction of one protein soluble in acid solution causes the loss of the other proteins soluble in basic solution, and vice versa as reported for peach allergens by Ahrazem et al [62].

Some allergens are present in very low concentration (e.g. allergen from Cypress pollen grains) or can be destroyed because of the enzymatic activity of the extract [63].

Some allergens, even though responsible for systemic reactions, can be absent from the allergenic extracts, as reported for hazelnut LTP [64].

Other disadvantages in using allergenic extracts are the possible batch to batch variations in protein and allergen concentrations, unless the preparations come from the same manufacturer. Thus, there is a lack quantification (i.e. μg/ml) of the allergenic proteins present in the extract [65,66].

Twenty-three allergenic proteins have been described in *Dermatophagoides pteronyssinus *(an allergenic source). However the presence and amount of all these proteins within any relevant extract are unknown. Very recently, the presence of some major allergens for some allergenic extracts has been stated by the pharmaceutical companies, but the presence of minor ones has never been reported, which might even be absent [64].

Lack of allergenic proteins or their low concentration could cause wrong negative diagnosis with consequent ineffective immunotherapy.

#### b) Presence of unwanted allergenic sources

During extraction procedures some relevant allergenic proteins can be lost, whereas others coming from different allergenic sources can contaminate the extract as they were present in the starting raw material used for the extraction. That is the case of some dog's allergenic extracts causing false positive ST in mite-sensitized patients because of the contamination of mite allergens in dog's epithelium, thus leading to a wrong diagnosis [67].

#### c) Perception of multiple sensitizations

The allergenic extracts do not allow the description of multiple sensitisations towards different allergens in patients with positive ST or specific IgE; they do not allow understanding if multiple sensitisation is due to IgE to distinct allergenic molecules (co-sensitization) or rather to a co-recognition (sensitization towards different allergenic sources, including the same allergenic molecules). As an example, a child suffering from food allergy, with a positive skin prick test to hazelnut extract might have a very different prognosis if the sensitization is linked to a Bet v 1-like protein or to a seed storage protein or to a lipid transfer protein. In the first case he does not run any risk of serious anaphylactic reaction, in the second and third case he should always carry auto-injectable epinephrine. In each case a different pattern of cutaneous reactivity could be recorded without being possible to understand the intimate sensitization profile. Moreover what said before is really a frustrating limit for the allergist, when deciding to prescribe a specific immunotherapy or an elimination diet in case of food allergy.

However, a patient characterized by allergy to a source might not show any symptoms towards cross reacting allergens, even in case of positive ST or specific IgE for those allergens is present. The reason for this phenomenon is not known yet [23,24,68].

### Advantages in using molecular allergens during the paediatric age

The number of purified recombinant or natural molecular allergens is in rapid increase, allowing to acquire more and more comprehensive and detailed information on child's sensitization profile [69].

Today it is possible, and it will be even easier in the future, to develop peptide sequences (peptide-chips) to describe the ultrafine IgE reactivity pattern of each patient. All these information will provide the overall and in depth description of the specific and antigenic features of the involved epitopes. The knowledge of the whole pattern of IgE responses will help to better understand the pathogenesis of allergic disease [69,70].

The application of micro-technology [14] and molecular allergens is leading to new methods for studying single patient's IgE co-recognition of homologous allergens [71,72].

Molecular diagnosis allows us to look forward the availability of a new specific immunotherapy, not based on undefined extracts anymore, but tailored on the single patient sensitization.

So far, not all allergenic sources have been completely characterized, and therefore the molecular diagnosis is not able to completely replace the allergenic extracts yet. All the different methods must complement and complete each other [15].

Knowledge of true offender protein contained in a food or in an inhalant allergenic source and relevant physico-chemical properties can allow primary and secondary prevention during the paediatric age. Should a child suffer from food anaphylaxis due to a certain protein, we could first of all prevent a new reaction by excluding from the diet not only that kind of food but also all the allergenic sources containing homologous proteins [73-75]. As a second step, some of the excluded foods containing homologous molecules having a low level of amino acid identity could then be reintroduced after specific challenge tests, to be carried out in a controlled setting. This could be done in a better and safer way if we can also test the molecule panel of interest by either ST or specific IgE reactivity. Moreover, taking into account the protein structural changes induced by heat and digestion, tolerance to cooked egg might be tested by some children allergic to raw egg [76], whereas tolerance to well done steak might be shown by some children allergic to underdone meat [7]. This could be easily reached when sensitization to heat labile allergenic epitopes is diagnosed. As a final example we can mention the case of a child suffering from oral allergic syndrome induced by nuts. As many children do, he is fond of Nutella, a delicious Italian hazelnut spread. Allowing or forbidding Nutella to such a child can be decided only upon exact knowledge of his allergenic molecule profile. In case of sensitization to Bet v 1-like proteins, or to profilins, Nutella might be allowed, whereas in case of sensitization to seed storage protein or to lipid transfer protein Nutella must be totally forbidden [77]. All above examples are not trivial, as they demonstrate the relevance of an appropriate planning of a correct diet for an allergic child, mainly in case of poly-sensitization. Paediatricians have to provide patients not only with the essential nutrients, but also with an optimised quality of life. As a matter of fact, useless forbiddances could only add marginalisation in a child or even rejection in a young adolescent. Furthermore, it is well known that asthma begins in childhood [78,79] and a good asthma prevention in a sensitized child might be reached by allergen detection and avoidance of true relevant exposure [80]. This can hold true for all the allergic diseases where allergen avoidance can be applied.

## Conclusion

The diagnosis using allergen extracts can be helpful identifying sensitization to a particular allergen source, but cannot resolve the molecular identity of the disease-eliciting allergen.

On the opposite, allergenic molecules allow the definition of a more precise patient's sensitization profile: if he or she is allergic to a genuine allergen, to major or minor determinant, or to a cross-reactive protein. Nowadays it is no more possible to test a patient with a huge number of allergenic extracts and feel satisfied because "...*those are the extracts commonly recognized by the majority of the population*". We still know too little to establish such limits, and there is no correlation between what is measured by epidemiological studies and what happens in a single patient. A single patient might also be a rare case (case report from the literature) but that rare case, that rare allergy, still represents 100% of the life of that patient, and of the diagnosis of that allergist. The rarity is also proportional to the prevalence of a measured phenomenon. A multiplex test using recombinant or natural purified allergenic molecules allows testing many allergens, with low costs and low serum volume required. This approach let reaching an insight as broad as possible on all the allergens recognized by a single patient.

Paediatrics is a branch of medical care dealing with assistance of infant, child, adolescent. The upper age limit ranges from age 14 to 18, depending on the country. Metabolism and immune system are age dependent. Infants, children, adolescents all live in the adults world, all breath the same allergenic molecules, all eat the same food allergens and at any time of their life they might become allergic. Until now no study has been performed to catch the sensitizing moment or to explain why an allergen is "stronger" than another one. A better understanding of these matters will be possible by following large paediatric population for years and searching for sensitizations by means of a multiple allergen array.

From all the above, it should be evident why even a non-allergist paediatrician needs to acquire the basic tools of this new knowledge. It is fundamental to continue to follow and correctly advise the little patients, staying in step with the times.

## Abbreviations

LTP: Lipid Transfer Protein

## Competing interests

The authors declare that they have no competing interests.

## Authors' contributions

CA and AM drafted the review manuscript; all authors revised and approved the final version.

## Appendix 1

Isoallergen and Variant Definitions

### Isoallergen

Similar molecules may be contained in a single allergenic source. They are named isoallergens when they divide similar molecular size, identical biologic function, if known, (e.g., enzymatic action), ≥ 67% identity of amino acid sequences.

Nevertheless, the recommended 67% identity in aminoacid sequence (, AllergomeBlaster tool [18]) is only a guide with a lot of borderline cases[21].

### Variants

Nucleotide mutations, either silent or leading to single or multiple amino acid substitutions can appear in the complementary DNA cloning of allergens causing single or multiple substitutions of aminoacids which generate variants. Therefore each isoallergen presents multiple forms of the same similar sequence, defined variants (identity greater than 95%).

Isoallergens and their variants belonging to the same allergen group are designated by suffixes of a period followed by four Arabic numerals. The first two numerals, 01 to 99, refer to a particular isoallergen; the two subsequent numerals, 01 to 99, refer to a particular variant of a particular isoallergen designated by the preceding two numerals e.g. Bet v 1.01 01 [21].
